# ﻿Two new species of the genus *Smaragdina* Chevrolat, 1836 (Coleoptera, Chrysomelidae, Cryptocephalinae) from China

**DOI:** 10.3897/zookeys.1082.74323

**Published:** 2022-01-18

**Authors:** Wen-Yuan Duan, Feng-Yan Wang, Hong-Zhang Zhou

**Affiliations:** 1 Key Laboratory of Zoological Systematics and Evolution, Institute of Zoology, Chinese Academy of Sciences, 1 Beichen West Rd, Chaoyang District, Beijing 100101, China Institute of Zoology, Chinese Academy of Sciences Beijing China; 2 University of the Chinese Academy of Sciences, 19A Yuquan Rd, Shijingshan District, Beijing 100049, China University of the Chinese Academy of Sciences Beijing China

**Keywords:** Clytrini, distributional records, leaf beetles, taxonomy

## Abstract

Two new species of the genus *Smaragdina* Chevrolat from China are reported: *S.hejingensis* Duan, Wang & Zhou **sp. nov.** from Xinjiang, and *S.magnipunctata* Duan, Wang and Zhou **sp. nov.** from Yunnan. Six species, *S.divisa* (Jacoby), *S.insulana* Medvedev, *S.kimotoi* Lopatin, *S.laboissierei* (Pic), *S.laosensis* Kimoto & Gressitt, and *S.oculata* Medvedev are new country records for China. Color illustrations and line drawings of general habitus and morphological details are given. All types of two new species are deposited in the collection of Institute of Zoology, Chinese Academy of Sciences (IZ-CAS).

## ﻿Introduction

The genus *Smaragdina* Chevrolat, 1836 has 350 recognized species (Warchałowski 2012; Wang and Zhou 2013), making it the most species-rich genus of the tribe Clytrini. The genus is distributed in the Palearctic, Oriental, and Afrotropical regions, with 64 species found in China (Clavareau 1913; Gressitt and Kimoto 1961; Tan et al. 1980; Kimoto and Gressitt 1981; Tan 1988; Lopatin 2004; Medvedev 2010; Regalin and Medvedev 2010; Warchałowski 2010, 2012; Wang and Zhou 2013). This genus is similar to *Aetheomorpha* Lacordaire, 1848 but can be distinguished by the slight epipleural lobe of elytra and the pygidium covered by the elytra (Warchałowski 2012). This distinction must be carefully used and examined, otherwise misidentification may occur for the beginner when facing some intermediate forms.

The Chinese species of *Smaragdina* have been studied by many specialists (e.g., Gressitt and Kimoto 1961; Tan et al. 1980; Kimoto and Gressitt 1981; Lopatin and Konstantinov 2009; Regalin and Medvedev 2010), but most of these studies were comprehensive and included all or most of the family Chrysomelidae, and *Smaragdina* was treated as only a part in those broader studies. Some recent studies on *Smaragdina* have included excellent keys to the species from the Oriental and Palearctic regions and, thus, are very important in identifying the Chinese species (Medvedev 2010; Warchałowski 2010). The most recent study on *Smaragdina* was by Wang and Zhou (2013), which is part of the series of publications concentrated on the Chinese leaf beetle fauna (Wang and Zhou 2011, 2012, 2013, 2020; Su and Zhou 2017; Duan and Zhou 2021; Duan et al. 2021a, 2021b).

There were few studies strictly concentrated on the biology of *Smaragdina*, i.e. life cycles, development, host plants, etc. Gao et al. (2019) studied the life cycle of *Smaragdinanigrifrons* (Hope, 1843) as vineyard pest, but this species is now moved out of the genus *Smaragdina*. Erber (1968, 1969, 1988) comprehensively reviewed Clytrinae and other related subfamilies, Agrain et al. (2015) reviewed the ant-nest related leaf beetles, and Chaboo et al. (2016) catalogued all known immature stages of camptosomate leaf beetles. Tan et al. (1980) provided some valuable records for host plants.

This paper, as a continuation of our leaf beetle studies (see above), describes two new species from China and reports six species as new country records for China. We increase the number of the Chinese species of *Smaragdina* to 72. We provide color illustrations and line drawings of general habitus and other structures for each species included. All types of the new species are deposited in the collection of Institute of Zoology, Chinese Academy of Sciences (IZ-CAS).

## ﻿Material and methods

### ﻿Dissection of specimens

Dried specimens were relaxed in hot distilled water at 80 °C for about 2 h to soften the body and ease dissection. The abdomen was separated with insect pins from the rest of the body and soaked in 10% KOH solution, and then in a hot water bath for 15 min to advance the process. After this procedure, the specimens were transferred to distilled water to rinse the residual KOH solution off and stop the bleaching process. The aedeagus, spermatheca, and rectal sclerites were dissected out from abdomen and placed in glycerin on a microslide.

### ﻿Photography and drawings

The dissected parts were placed into glycerin for observation and measured with an apochromatic stereomicroscope Zeiss SteREO V12. Color photographs of the adults and genitalia were captured with an Axio Zoom V16 fluorescence stereo zoom microscope and photomontage was performed in Zen 2012 (blue edition) imaging software. Adobe Photoshop CS6 was used in digital post-processing of the color images, and Adobe Illustrator 2020 was used to make the line drawings.

### ﻿Morphological terminology and measurement

The term “rectal sclerites” (ventral rectal sclerites, dorsal rectal sclerites) is used throughout the paper according to Schöller et al. 2008, and “fossa” is used throughout the paper to indicate the round and deep structure on the last abdominal segment in female; it is widely used term in many publications in Cryptocephalinae (Gressitt and Kimoto 1961; Tan et al. 1980; Kimoto and Gressitt 1981; Tan 1988). Body length was measured to cover the whole length from the apex of pronotum to the apex of elytra in dorsal view.

### ﻿Specimens studied

Specimens used in this study are from the Institute of Zoology, Chinese Academy of Sciences, Beijing, China (**IZ-CAS**).

Measurements are average values calculated from the values of at least 10 specimens, or all if fewer specimens were available. The following abbreviations were used to identify the institutions of holotype deposition.

**BPBM**Bernice P. Bishop Museum, Honolulu, Hawaii, USA;

**LM** Lev N. Medvedev Collection, Moscow, Russia;

**MNHN**National Museum of Natural History, Paris, France.

### ﻿Host plants for Chinese *Smaragdina* species (from Tan et al. 1980)

*Smaragdinamandzhura*: Gramineae, *Miscanthus*

*Smaragdinasemiaurantiaca*: Rosaceae, *Prunus*, *Malus*

*Smaragdinaauritahammarstroemi*: Salicaceae, *Salix*, *Populus*

*Smaragdinamandzhura*: Ulmaceae, *Ulmus*

*Smaragdinasemiaurantiaca*: Ulmaceae, *Ulmus*

*Smaragdinamandzhura*: Rhamnaceae, *Ziziphusjujuba*

*Smaragdinaauritahammarstroemi*: Betulaceae, *Betula*; Melastomataceae, *Styraxjaponica*

## ﻿Results

### ﻿Taxonomy

**Diagnosis.** Body shape elongate and subcylindrical, usually smaller than 6 mm. Head small, very short; mandibles short; eyes round or elongate; antennae slender, 2^nd^ and 3^rd^ antennomeres short and equal, following antennomeres serrate. Pronotum transverse, posterior angles rounded; scutellum large. Elytra without distinct epipleural lobes. Legs short, fore legs sometimes slightly longer than others; tarsi of females usually long and narrow, 1^st^ tarsomere longer than 2^nd^; legs of males usually stouter than female’s, tarsi broader and shorter. Pygidium not exposed.

#### ﻿ Smaragdina hejingensis

Taxon classificationAnimaliaColeopteraChrysomelidae

Duan, Wang & Zhou sp. nov.

CBF72377-8C73-5475-8202-6B51F06EBD35

http://zoobank.org/24F5ED4A-681C-4D20-A8C4-CD404D4F8D40

Figures 1 , 2

##### Diagnosis.

This new species is the nearest to *S.flavilabris* (Briet, 1917) but can be distinguished by the shape of pronotal marking: *S.flavilabris* has the metallic blue marking rhombic, whereas in the new species this marking is not. Additionally, the anterior margin of the clypeus in the new species is yellowish brown but black in *S.flavilabris*.

##### Etymology.

The specific is named after the type locality, Hejing.

##### Type locality.

China: Xinjiang: Hejing.

##### Material examined

**(*n* = 6). *Holotype***: male, **China: Xinjiang**: Hejing, 30.VII.1958, coll. Changqing Li (IZ-CAS). ***Paratypes*: China: Xinjiang**: 3 males, 2 females, Hejing, 31.VII.1958, coll. Changqing Li (IZ-CAS).

##### Measurements

**(*n* = 6).** Body length males: 5.2–5.9 mm, females: 5.1–5.6 mm.

##### Description.

**Figure 1.**
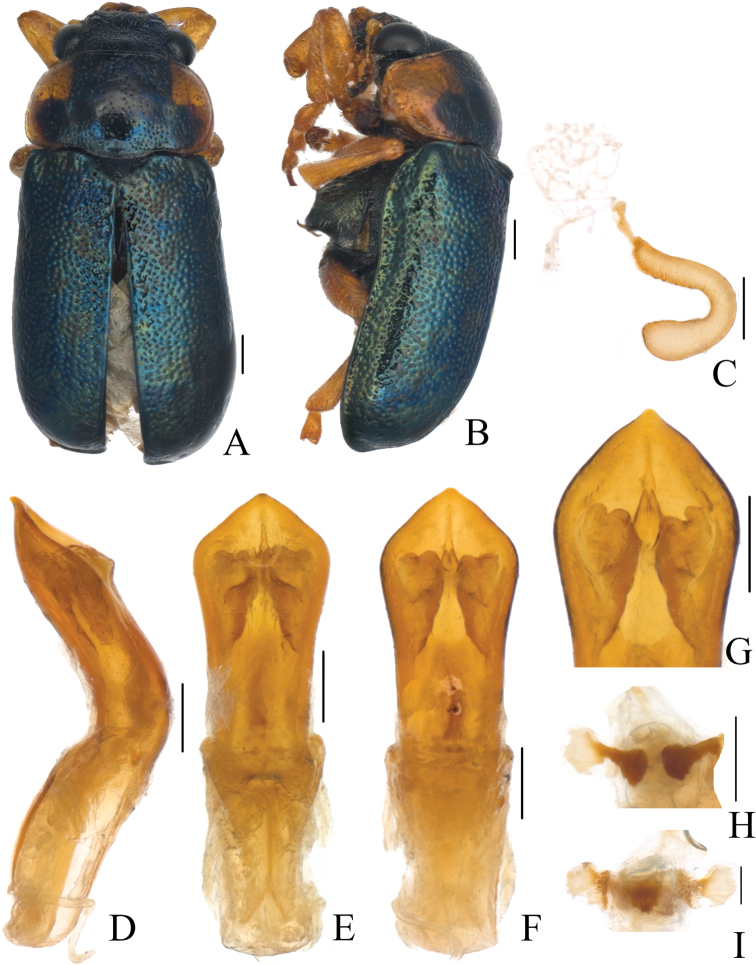
*Smaragdinahejingensis* Duan, Wang & Zhou, sp. nov. **A** habitus **B** lateral view of habitus **C** spermatheca **D** lateral view of aedeagus **E** ventral view of aedeagus **F** dorsal view of aedeagus **G** apex of aedeagus **H** ventral rectal sclerites **I** dorsal rectal sclerites. Scale bars: 0.5 mm (**A, B**), 0.2 mm (**C–I**).

**Figure 2.**
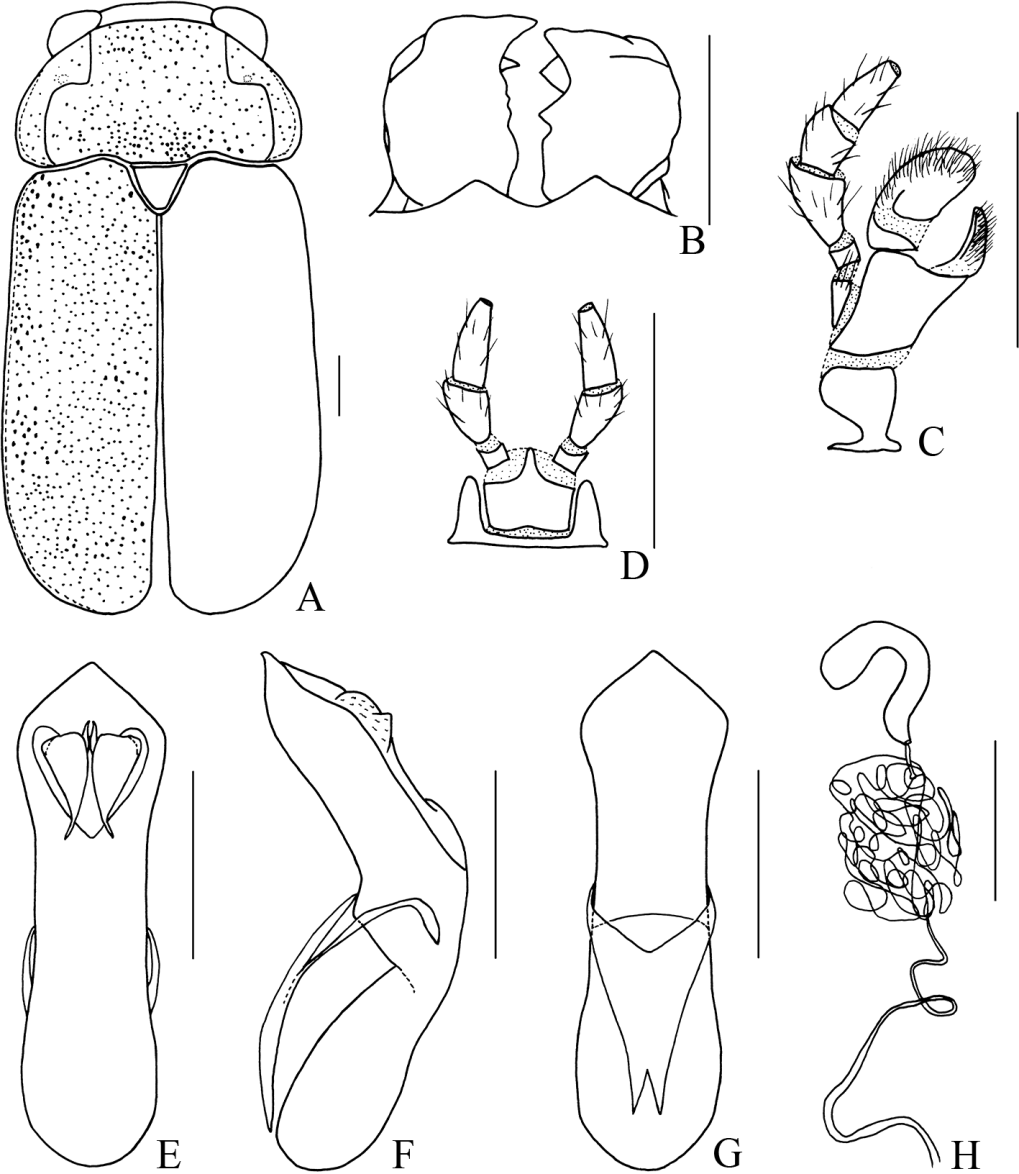
*Smaragdinahejingensis* Duan, Wang & Zhou, sp. nov. **A** habitus **B** mandibles **C** maxilla **D** labium **E** dorsal view of aedeagus **F** lateral view of aedeagus **G** ventral view of aedeagus **H** spermatheca. Scale bars: 0.5 mm.

Body (Figs 1A, B, 2A) oblong, largely metallic blue. Mandibles yellowish brown, apex tinged with brown; labrum darkish brown; ventral side of mouthpart yellowish brown; four basal antennomeres yellow, others brown. Pronotum largely metallic blue, lateral sides with yellowish brown marking. Scutellum black. Ventral side of body largely metallic blue, prosternum yellowish brown, femora of hind legs tinged with black, last segments of tarsi and claws brown.

Head small, with dense and coarse punctures. Mandibles (Fig. 2B) short, labrum slightly incised at anterior margin, length ratio of maxillary palpomeres 0.5:2.4:1.5:2.4; while that of labial palpomeres 0.5:1.8:2.8; mentum rectangular emarginated at anterior margin. Clypeus lateral sides slightly depressed, anterior margin feebly incised; frons covered with shallow wrinkles; inner sides of eyes with short pubescence; vertex not convex, with fine wrinkles. Antennae short, extending to base of prothorax, pubescent, 1^st^ antennomere oblong, 2^nd^ rounded, 3^rd^ slender, slightly longer than 2^nd^, 4^th^ triangular, a little longer than 3^rd^, serrated from 5^th^ segment onwards.

Pronotum transverse, about 2× as wide as long, moderately convex; anterior margin slightly convex, lateral margins rounded, posterior margin sinuated, all margins (especially lateral ones) bordered; surface with coarse punctures, denser on median basal area. Scutellum widely triangular, with extremely fine punctures; apex slightly elevated over elytral surface.

Elytra 1.7 times as long as wide at humeri, covered with confused and dense punctures, interstices shorter than a puncture diameter, punctures becoming sparse posteriorly, and nearly disappearing at elytral apices.

Underside and legs thickly clothed with silvery pubescence; apex of pygidium arcuate. Tarsi slender, length ratio of protarsomeres 1.6:1.0:0.3:2.0. Tibiae of females slightly curved, legs robust, tarsi broad.

Aedeagus (Figs 1D–G, 2E–G) sword-shaped, about 3.4× as long as wide; apex triangular; bent ventrally; without pubescence.

**Female.** Coloration of body darker than male, base of hind tibiae black; legs slender, tarsi narrow. Last segment of abdomen with a fossa. Spermatheca (Figs 1C, 2H) hook-shaped, apex swollen and blunt, duct base thickened, not coiled. Rectal sclerites moderately sclerotized, ventral rectal sclerites (Fig. 1H) large, clubbed; dorsal rectal sclerites (Fig. 1I) protruding medially; lateral sclerites large.

##### Distribution.

China (Xinjiang).

#### ﻿ Smaragdina magnipunctata

Taxon classificationAnimaliaColeopteraChrysomelidae

Duan, Wang & Zhou sp. nov.

3C61B8F8-7104-549E-85FA-00BC0138B426

http://zoobank.org/DB4FD31E-D2CF-45DF-91C6-2A7CE6B84FA5

Figures 3 , 4

##### Diagnosis.

This new species is well distinguished from all its congeners by the presence of unique black markings on the elytra and strong punctures arranged in regular rows with their interspaces impunctate.

##### Etymology.

The specific epithet is from the Latin words “*magni*-” and “*punctata*” in reference to the big punctures of the elytra.

##### Type locality.

China: Yunnan Province: Xishuangbanna, Xiaomengyang.

##### Material examined

**(*n* = 4). *Holotype***: male, **China: Yunnan Province**: Xishuangbanna, Xiaomengyang, 7.VII.1957, coll. Shuyong Wang (IZ-CAS). ***Paratypes*: China: Yunnan Province**: 1 female, Xishuangbanna, Xiaomengyang, 10.X.1957, coll. Shuyong Wang (IZ-CAS); 1 female, Xishuangbanna, Xiaomengyang, 21.X.1957, coll. Shuyong Wang (IZ-CAS); 1 female, Xishuangbanna, Xiaomengyang, 22.X.1957, coll. Lingchao Zang (IZ-CAS).

##### Measurements

**(*n* = 4).** Body length males: 3.7 mm, females: 3.9–4.3 mm.

##### Description.

**Figure 3.**
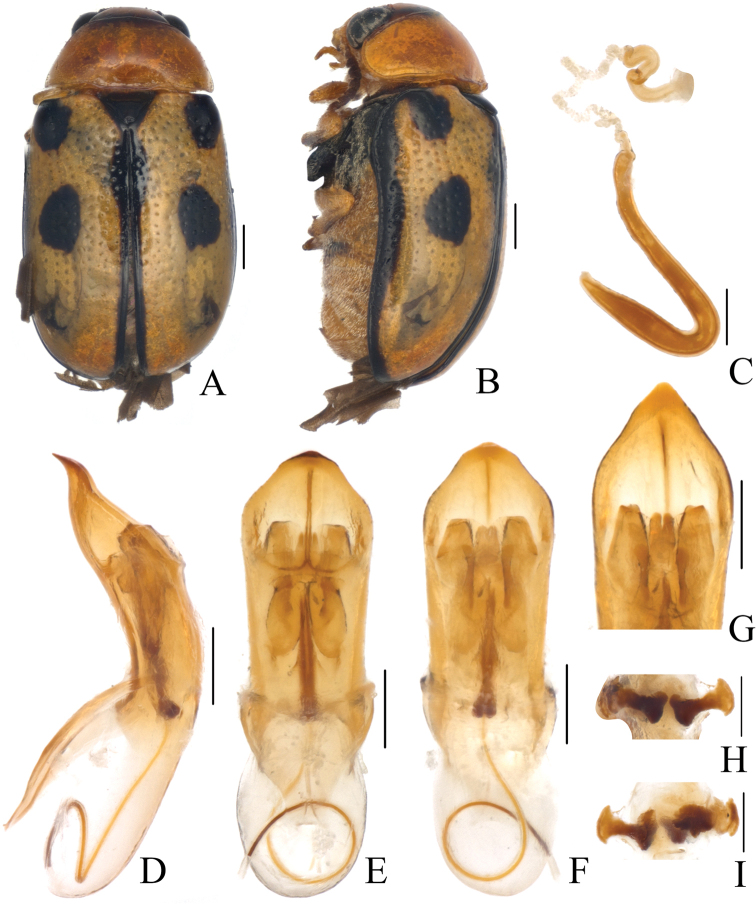
*Smaragdinamagnipunctata* Duan, Wang & Zhou, sp. nov. **A** habitus **B** lateral view of habitus **C** spermatheca **D** lateral view of aedeagus **E** ventral view of aedeagus **F** dorsal view of aedeagus **G** apex of aedeagus **H** ventral rectal sclerites **I** dorsal rectal sclerites. Scale bars: 0.5 mm (**A, B**), 0.2 mm (**C–I**).

**Figure 4.**
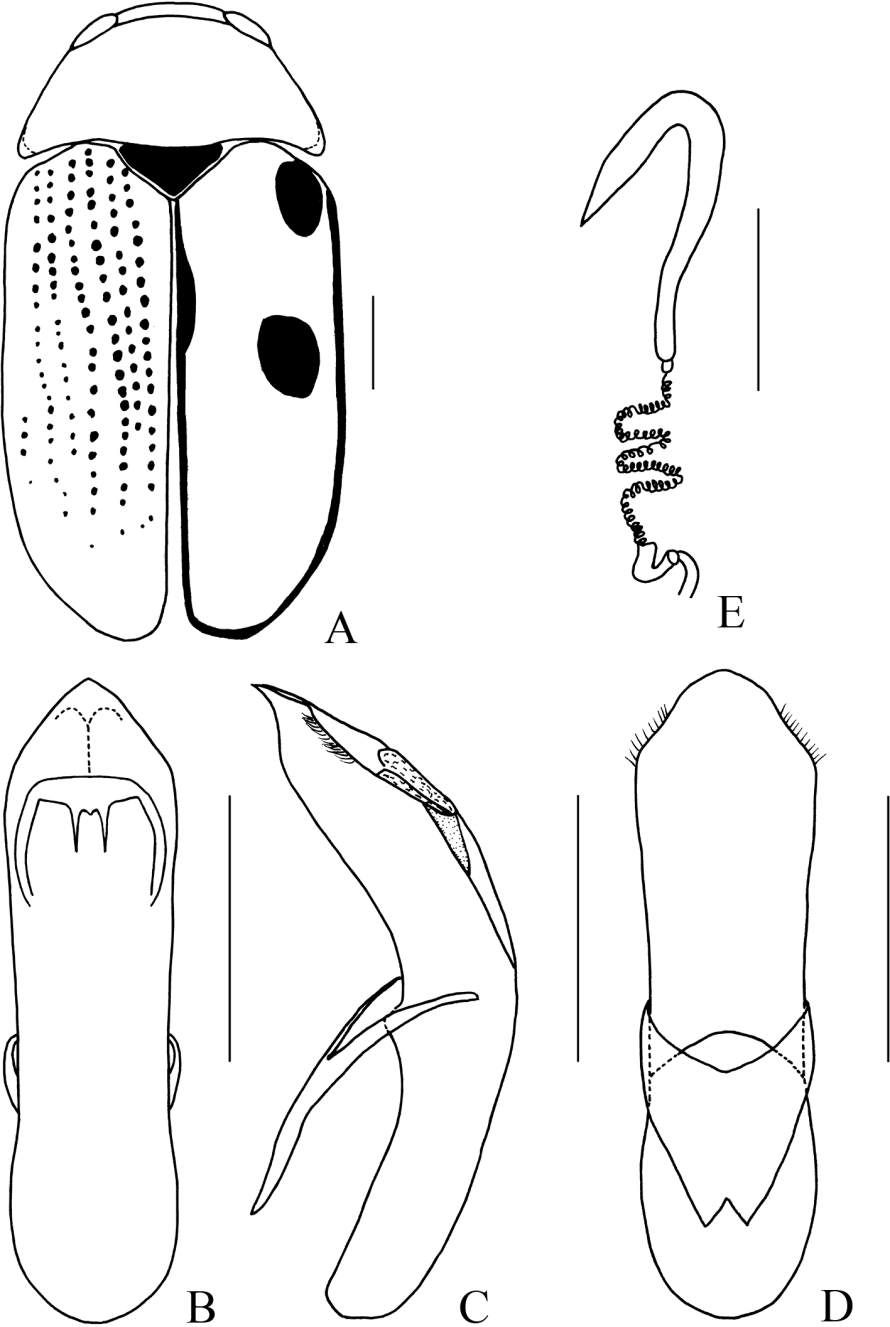
*Smaragdinamagnipunctata* Duan, Wang & Zhou, sp. nov. **A** habitus **B** dorsal view of aedeagus **C** lateral view of aedeagus **D** ventral view of aedeagus **E** spermatheca. Scale bars: 0.5 mm.

Body (Figs 3A, B, 4A) oblong, largely khaki. Head yellowish brown, Mandibles with black apex; two basal antennomeres yellow, others brown; sometimes vertex black. Pronotum yellowish brown. Scutellum black. Elytra khaki, margins black, disc with two black round markings. Ventral side of body largely yellowish brown, ventral side of mesothorax and metathorax black; legs yellowish, tibiae outer side and tarsi somewhat brown.

Head small, glabrous. Mandibles short; labrum slightly incised at anterior margin; length ratio of maxillary palpomeres 0.5:2.2:2.0:2.5; length ratio of labial palpomeres 0.4:1.8:3.0. Clypeus glabrous, anterior margin slightly incised; frons with 3 grooves arranged in triangular pattern; inner sides of eyes with short pubescence; vertex slightly convex, glabrous. Antennae short, extending to base of prothorax, pubescent, 1^st^ antennomere oblong and thick, 2^nd^ rounded, 3^rd^ slender, similar in length with 2^nd^, 4^th^ triangular, a little longer than 3^rd^, serrated from 5^th^ segment onwards, while last segment ovate, apex sharp.

Pronotum transverse, about 2× as wide as long, strongly convex; anterior and lateral margins rounded, posterior margin weakly sinuated and bordered; surface without any punctures. Scutellum triangular, impunctate, apex slightly elevated over elytral surface.

Elytra glabrous, 1.5× as long as wide at humeri; 2/3 basal part with coarse, sparse punctures, forming regular rows, with interspaces impunctate; 1/3 apical part with sparse, fine punctures, almost obsolete.

Underside and legs thickly clothed with silvery pubescence; apex of pygidium slightly concave. Tarsi slender, length ratio of protarsomeres 1.0:0.7:0.2:1.2.

Aedeagus (Figs 3D–G, 4B–D) oblong, about 3.6× as long as wide, apex triangular, bent ventrally; without pubescence.

**Female.** Punctures of elytra more obvious than male, last segment of abdomen with a fossa, apex of pygidium truncate. Spermatheca (Figs 3C, 4E) hooked, slender; duct strongly sclerotized, base thickened, coiled up about 20–30×. Rectal sclerites strongly sclerotized, ventral rectal sclerites (Fig. 3H) large, dumbbell-like; dorsal central sclerite (Fig. 3I) rectangular; lateral sclerites slightly small.

##### Distribution.

China (Yunnan).

#### ﻿ Smaragdina divisa

Taxon classificationAnimaliaColeopteraChrysomelidae

(Jacoby, 1889), new country record for China

B6D23E98-A00E-5CA1-8DE3-D7B21FD17A81

Figures 5 , 6

Smaragdina divisa Jacoby, 1889: 156 (orig.: Gynandrophthalmadivisa); 1908: 117, fig. 29; Clavareau 1913: 60; Medvedev 1988a: 31 (redescription); Medvedev 2010:265; Regalin and Medvedev 2010: 576 (catalogue).
Gynandrophthalma indica Jacoby, 1895: 263; Jacoby 1908: 117 (as synonym of Gynandrophthalmadivisa). Syn.

##### Material examined

**(*n* = 17). China: Hainan province**: 6 males, 4 females, Jianfeng, 20.IV.1980, coll. Fuji Pu (IZ-CAS); 2 males, Jianfenglin, 20.IV.1980, coll. unknown (IZ-CAS); 1 male, Lehui, 4.V.1954, coll. Keren Huang (IZ-CAS); 1 female, Nada, 25.IV.1954, coll. Keren Huang (IZ-CAS); 1 female, Baoting, 16.V.1960, coll. Zhenfu Li (IZ-CAS); 1 female, Ledong, 10.VI.1960, coll. Xuezhong Zhang (IZ-CAS); 1 female, Tongshi, 8.V.1960, coll. Zhenfu Li (IZ-CAS).

**Figure 5.**
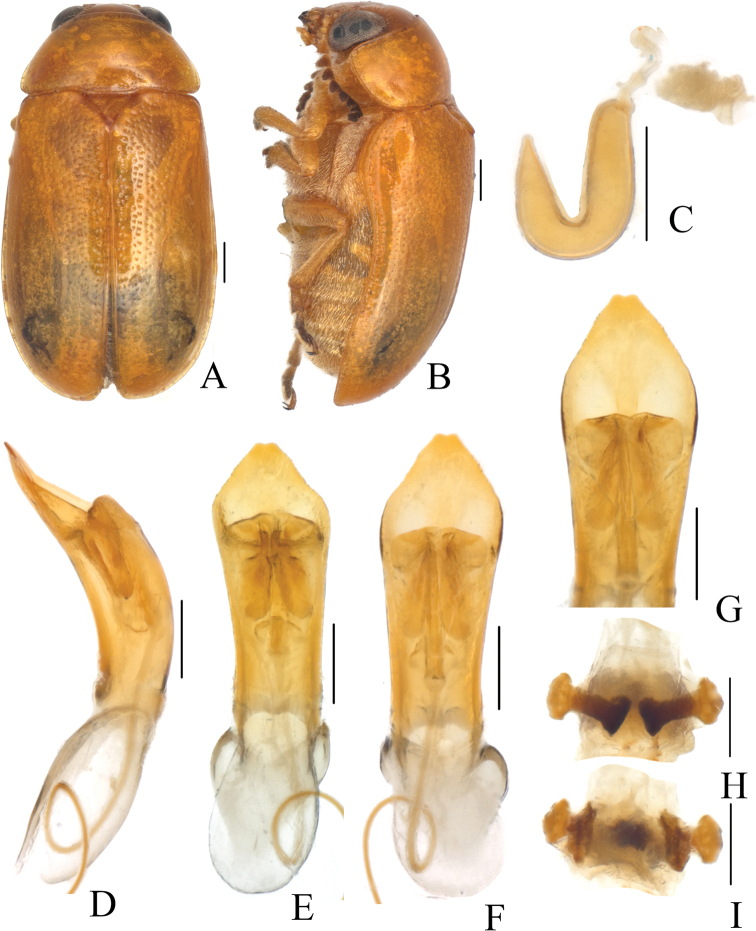
*Smaragdinadivisa* (Jacoby, 1889) **A** habitus **B** lateral view of habitus **C** spermatheca **D** lateral view of aedeagus **E** ventral view of aedeagus **F** dorsal view of aedeagus **G** apex of aedeagus **H** ventral rectal sclerites **I** dorsal rectal sclerites. Scale bars: 0.5 mm (**A, B**), 0.2 mm (**C–I**).

##### Measurements

**(*n* = 10).** Body length males: 3.4–3.8 mm, females: 5.1–5.3 mm.

**Figure 6.**
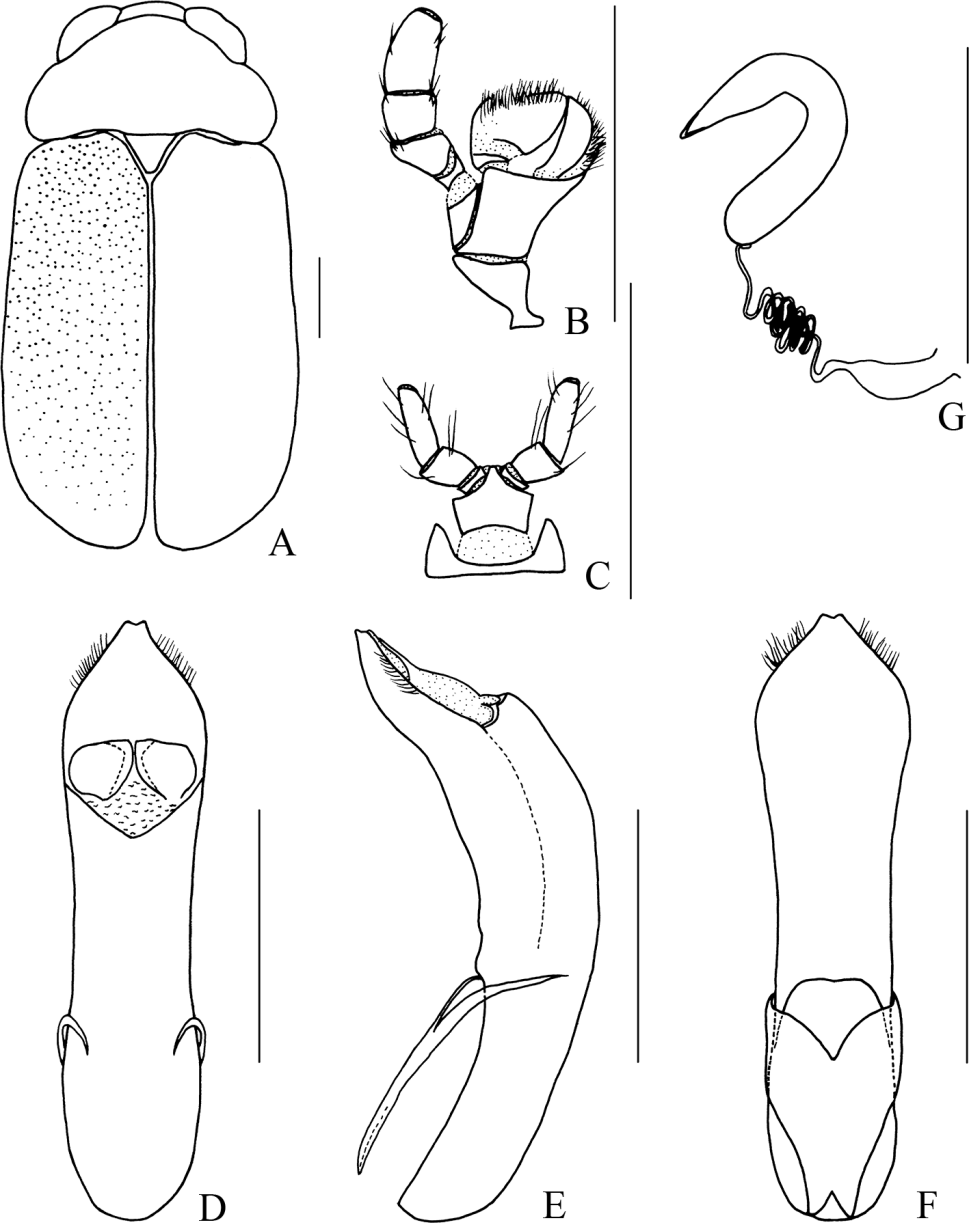
*Smaragdinadivisa* (Jacoby, 1889) **A** habitus **B** maxilla **C** labium **D** dorsal view of aedeagus **E** lateral view of aedeagus **F** ventral view of aedeagus **G** spermatheca. Scale bars: 0.5 mm.

##### Distribution.

China (Hainan); Vietnam; Burma; Nepal; Sri Lanka; Malaysia.

##### Remark.

This species is recognized by having the apical half of the elytra distinctly paler than the basal half, and the apex of aedeagus narrowly truncate. It has not previously been recorded from the territory of China.

#### ﻿ Smaragdina insulana

Taxon classificationAnimaliaColeopteraChrysomelidae

Medvedev, 1992, new country record for China

D61D5415-20B6-5190-ACFC-BF4565FAC1F5

Figures 7 , 8

Smaragdina insulana Medvedev, 1992a: 73 (type locality: Vietnam, Prov. Quang Nam-Da Nang); Medvedev 2010: 266.

##### Material examined

**(*n* = 3). China: Guangxi province**: 1 female, Longzhou, Daqingshan, 20.IV.1963, coll. Shuyong Wang (IZ-CAS); 1 male, Longzhou, Daqingshan, 26.IV.1963, coll. Shuyong Wang (IZ-CAS); 1 male, Longzhou, Daqingshan, 27.IV.1963, coll. Shuyong Wang (IZ-CAS).

**Figure 7.**
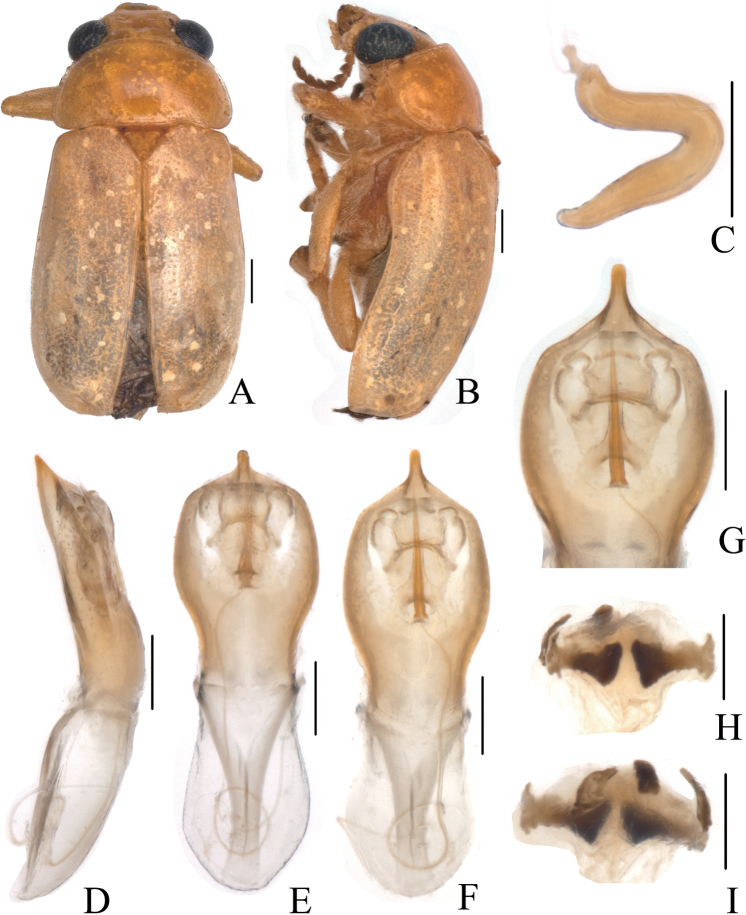
*Smaragdinainsulana* Medvedev, 1992 **A** habitus **B** lateral view of habitus **C** spermatheca **D** lateral view of aedeagus **E** ventral view of aedeagus **F** dorsal view of aedeagus **G** apex of aedeagus **H** ventral rectal sclerites **I** dorsal rectal sclerites. Scale bars: 0.5 mm (**A, B**), 0.2 mm (**C–I**).

##### Measurements

**(*n* = 3).** Body length males: 4.8–4.9 mm, females: 5.0 mm.

**Figure 8.**
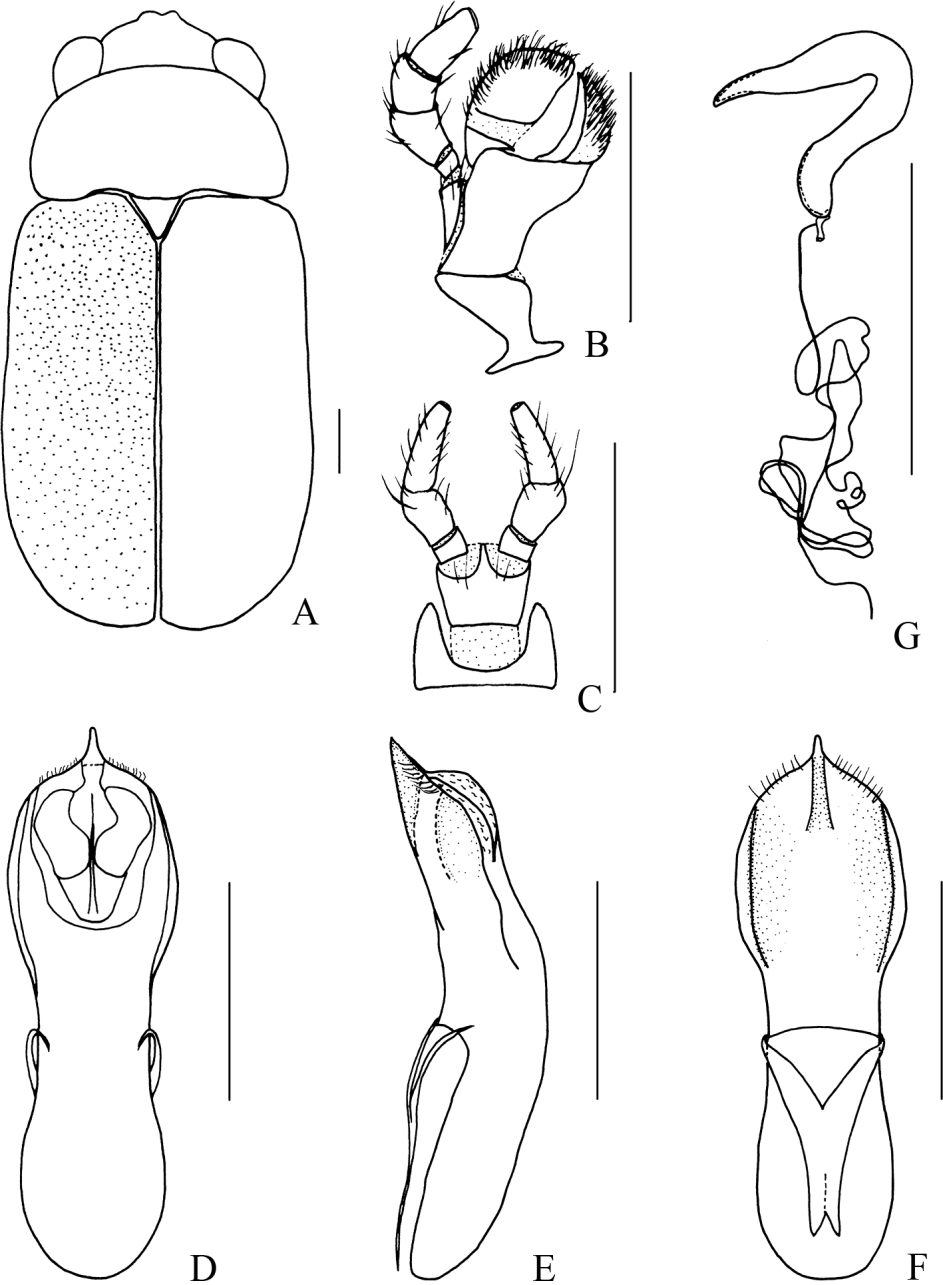
*Smaragdinainsulana* Medvedev, 1992 **A** habitus **B** maxilla **C** labium **D** dorsal view of aedeagus **E** lateral view of aedeagus **F** ventral view of aedeagus **G** spermatheca. Scale bars: 0.5 mm.

##### Distribution.

China (Guangxi); Vietnam.

##### Remark.

This species is recognized by the fulvous body color, pale flavous spots on the elytra, and the sharp apex of the aedeagus. Originally found in Vietnam, we can confirm this species occurs in China.

#### ﻿ Smaragdina kimotoi

Taxon classificationAnimaliaColeopteraChrysomelidae

Lopatin, 2003, new country record for China

A8998833-45DC-5A32-B56C-A40CC949F450

Figures 9 , 10

Smaragdina kimotoi Lopatin, 2003: 301 (type locality: South Vietnam, northeast of Ho Chi Minh); Medvedev 2010: 284.

##### Material examined

**(*n* = 13). China: Hainan province**: 1 male, 3 females, Jianfeng, 20.IV.1980, coll. Fuji Pu (IZ-CAS); 2 males, Jianfenglin, 29.IV.1983, coll. Maobin Gu (IZ-CAS); 1 female, Jianfenglin, 19.IV.1984, coll. Chunling Wang (IZ-CAS); 1 female, Wuzhishan, 4.IV.1980, coll. Shuyong Wang (IZ-CAS); 1 female, Nada, 25.IV.1954, coll. Keren Huang (IZ-CAS); 2 females, Nada, 30.IV.1954, coll. Keren Huang (IZ-CAS); 1 male, Nada, 30.V.1954, coll. Keren Huang (IZ-CAS); 1 female, Tongshi, 27.III.1960, coll. Changqing Li (IZ-CAS); 1 female, Tongshi, 23.IV.1960, coll. Zhenfu Li (IZ-CAS).

**Figure 9.**
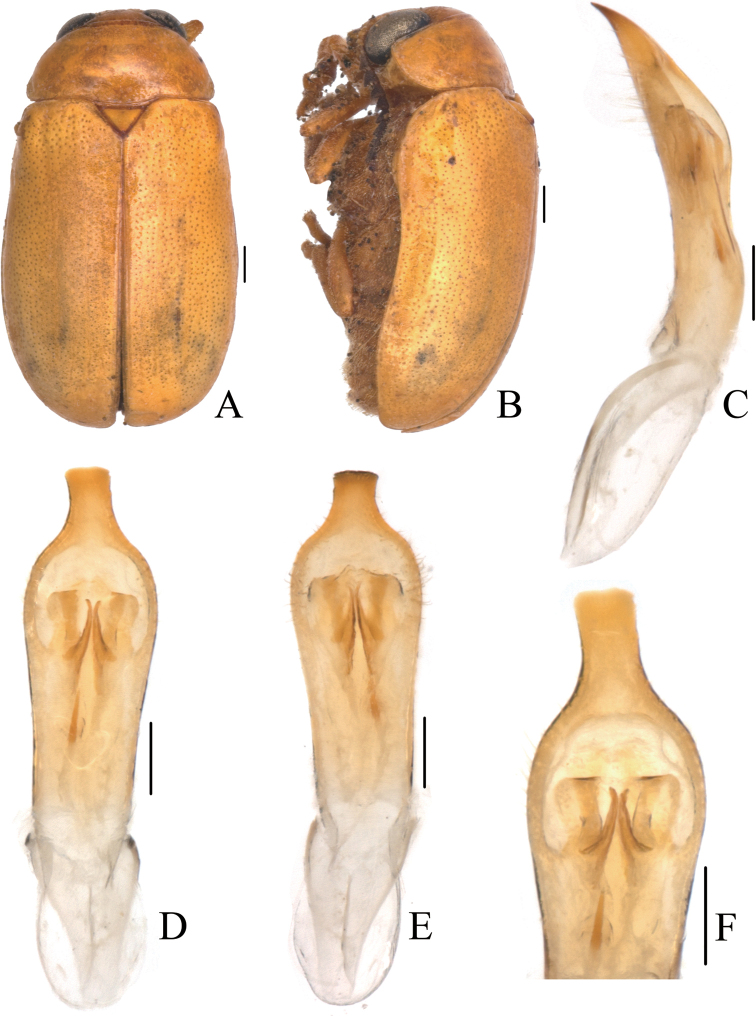
*Smaragdinakimotoi* Lopatin, 2003 **A** habitus **B** lateral view of habitus **C** lateral view of aedeagus **D** ventral view of aedeagus **E** dorsal view of aedeagus **F** apex of aedeagus. Scale bars: 0.5 mm (**A, B**), 0.2 mm (**C–F**).

##### Measurements

**(*n* = 10).** Body length males: 4.4–4.9 mm, females: 4.9–5.9 mm.

**Figure 10.**
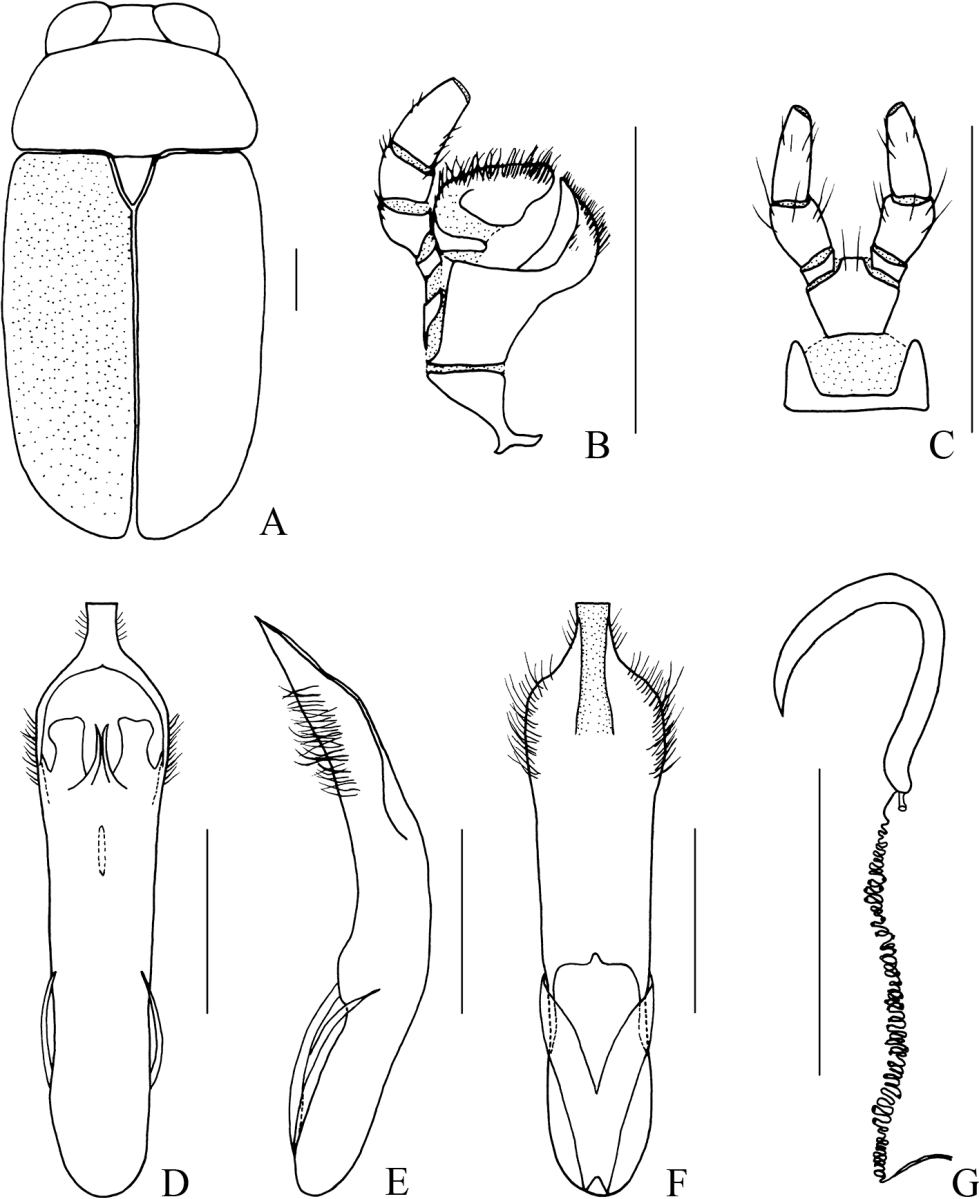
*Smaragdinakimotoi* Lopatin, 2003 **A** habitus **B** maxilla **C** labium **D** dorsal view of aedeagus **E** lateral view of aedeagus **F** ventral view of aedeagus **G** spermatheca. Scale bars: 0.5 mm.

##### Distribution.

China (Hainan); Vietnam.

##### Remark.

The fulvous dorsal body and the aedeagus with a broad and parallel-sided apical process, distinguish this species from *S.divisa*. Originally from Vietnam, we confirm that *S.kimotoi* occurs in China.

#### ﻿ Smaragdina laboissierei

Taxon classificationAnimaliaColeopteraChrysomelidae

(Pic, 1928), new country record for China

30FB570B-0BB8-583F-AF6E-DB351C211615

Figures 11 , 12

Smaragdina laboissierei Pic, 1928: 34 (orig.: Cyanirislaboissierei; type locality: Tonkin; type deposited: MNHN); Kimoto and Gressitt 1981: 320 (Smaragdinalaboissierei); Medvedev 1992b: 23 (figure); Medvedev 2010: 264.

##### Material examined

**(*n* = 2). China: Guangxi province**: 1 female, Longzhou, Daqingshan, 19. IV. 1963, coll. Shuyong Wang (IZ-CAS); 1 male, Longzhou, Daqingshan, 27. IV. 1963, coll. Shuyong Wang (IZ-CAS).

**Figure 11.**
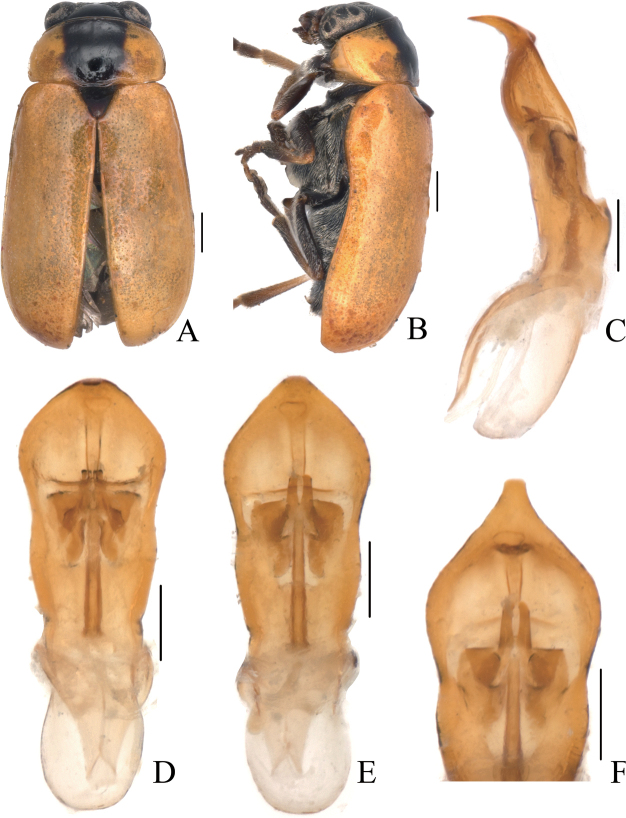
*Smaragdinalaboissierei* (Pic, 1928) **A** habitus **B** lateral view of habitus **C** lateral view of aedeagus **D** ventral view of aedeagus **E** dorsal view of aedeagus **F** apex of aedeagus. Scale bars: 0.5 mm (**A, B**), 0.2 mm (**C–F**).

##### Measurements

**(*n* = 2).** Body length males: 4.4–4.7 mm, females: 5.2–5.5 mm.

**Figure 12.**
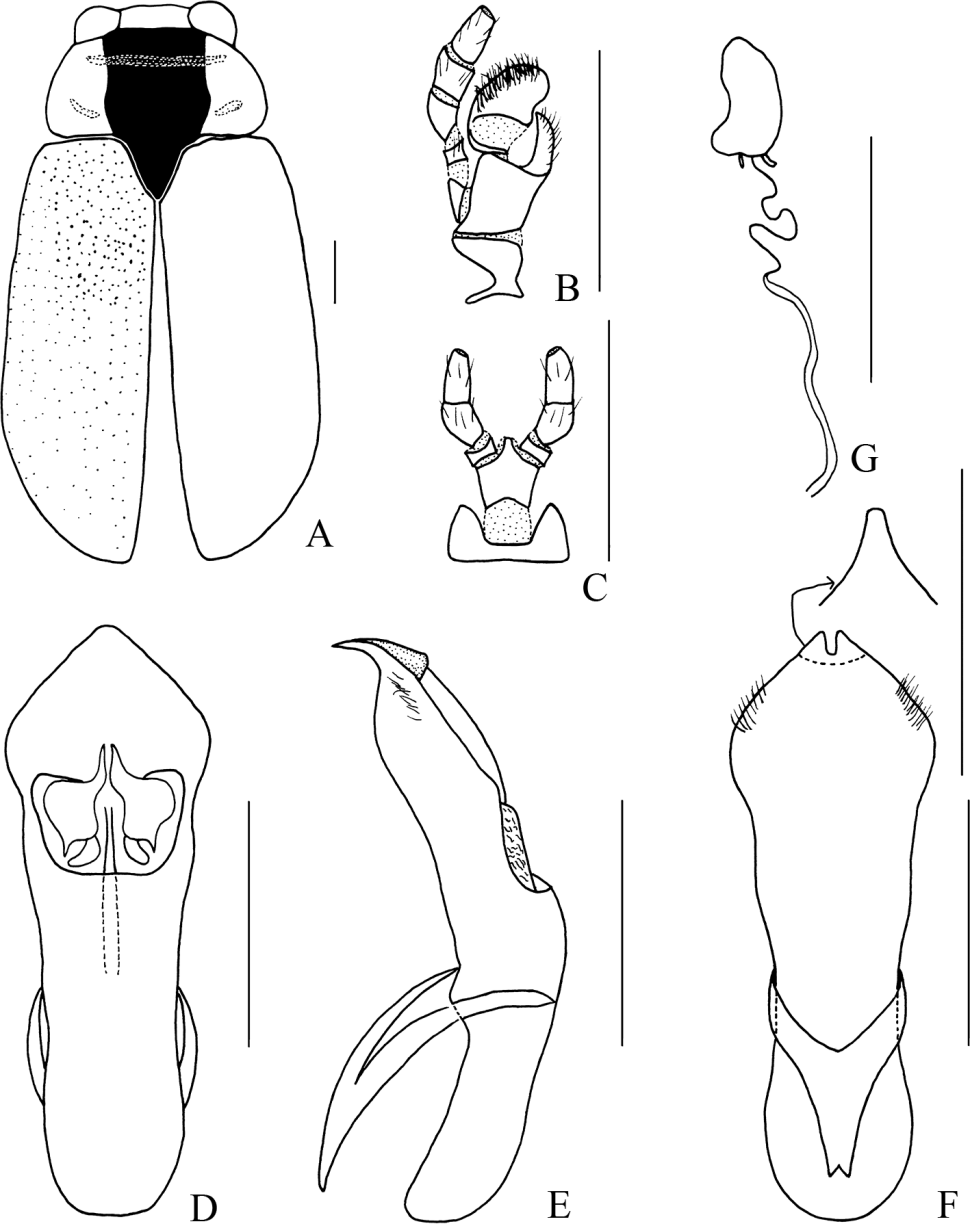
*Smaragdinalaboissierei* (Pic, 1928) **A** habitus **B** maxilla **C** labium **D** dorsal view of aedeagus **E** lateral view of aedeagus **F** ventral view of aedeagus **G** spermatheca. Scale bars: 0.5 mm.

##### Distribution.

China (Guangxi); Vietnam.

##### Remark.

The Chinese specimens were identified as this species by the yellowish-brown pronotum with a black stripe and the apical process of aedeagus triangular, which is acute and strongly curved downwards. This species was previously only known from Vietnam.

#### ﻿ Smaragdina laosensis

Taxon classificationAnimaliaColeopteraChrysomelidae

Kimoto & Gressitt, 1981, new country record for China

76012F10-B288-5B60-8E06-64958888645D

Figures 13 , 14

Smaragdina laosensis Kimoto & Gressitt, 1981: 320 (type locality: Laos; type deposited: BPBM); Medvedev 2010: 269.

##### Material examined

**(*n* = 5). China: Yunnan province**: 1 female, Cheli, 8.III.1957, coll. Fuji Pu (IZ-CAS); 1 male, Cheli, 11.III.1957, coll. Fuji Pu (IZ-CAS); 2 males, Xishuangbanna, Yunjinghong, 3.IV.1958, coll. unknown (IZ-CAS); 1 female, Xishuangbanna, Yunjinghong, 7.VIII.1958, coll. Xuwu Meng (IZ-CAS).

**Figure 13.**
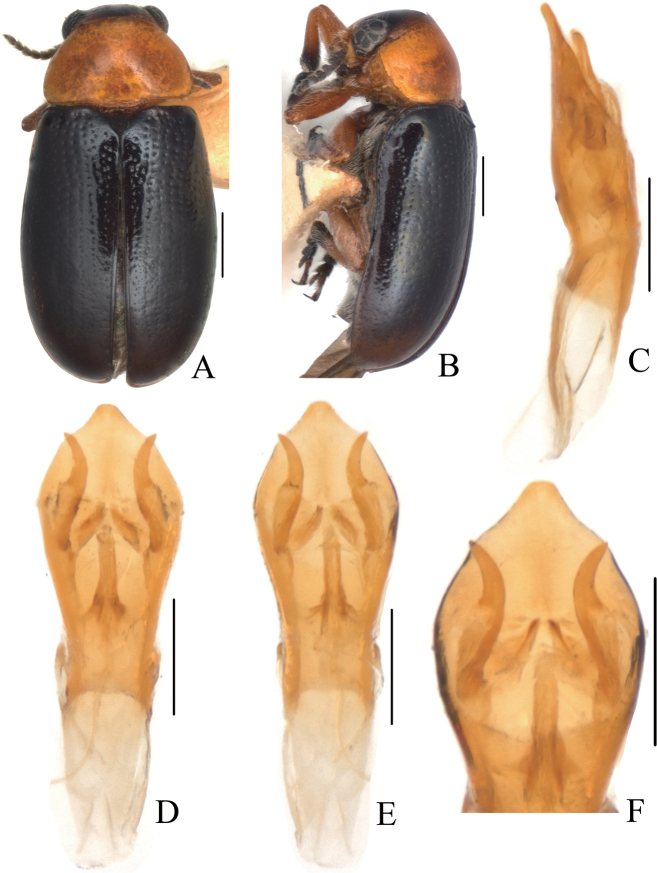
*Smaragdinalaosensis* Kimoto & Gressitt, 1981 **A** habitus **B** lateral view of habitus **C** lateral view of aedeagus **D** ventral view of aedeagus **E** dorsal view of aedeagus **F** apex of aedeagus. Scale bars: 0.5 mm (**A, B**), 0.2 mm (**C–F**).

##### Measurements

**(*n* = 5).** Body length males: 2.9–3.2 mm, females: 3.3–3.5 mm.

**Figure 14.**
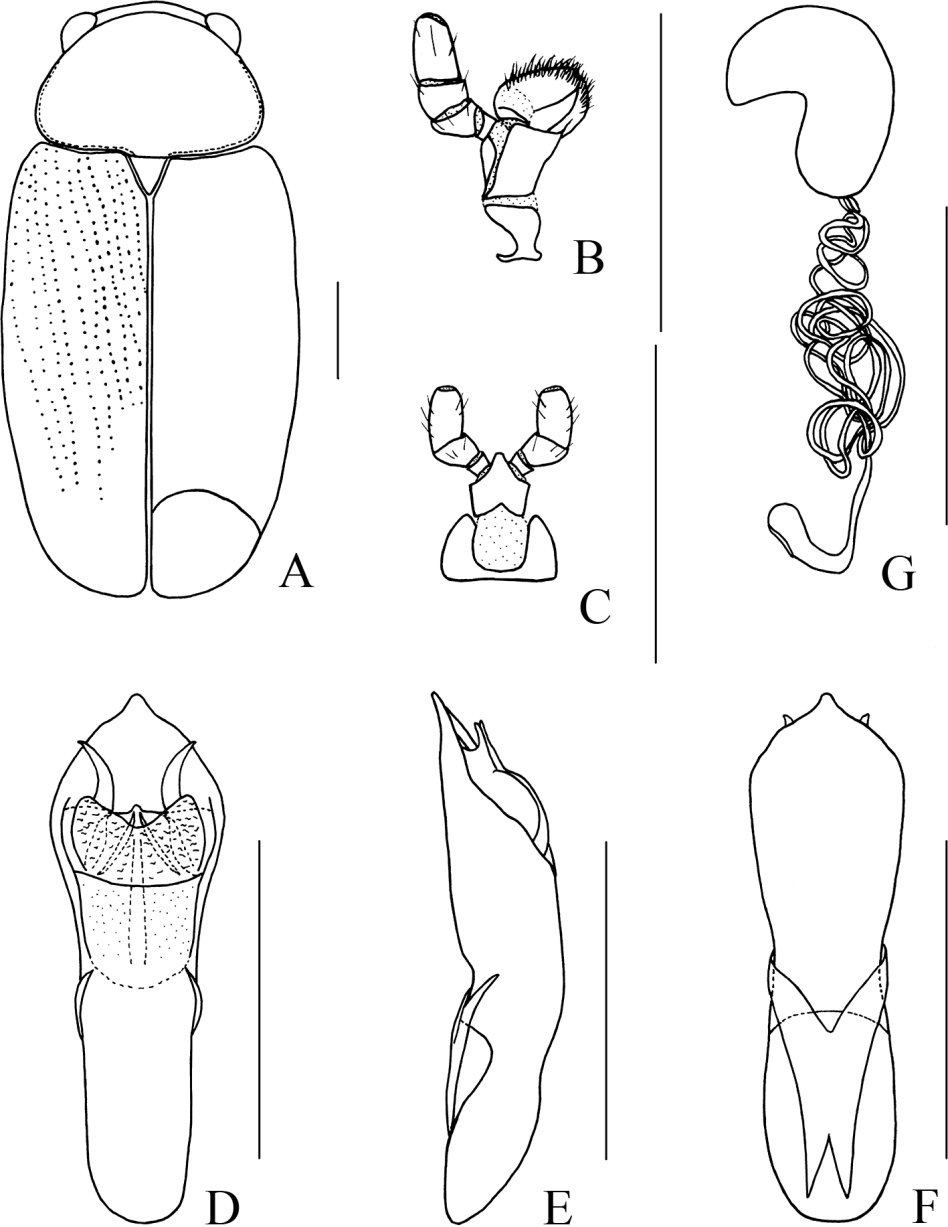
*Smaragdinalaosensis* Kimoto & Gressitt, 1981 **A** habitus **B** maxilla **C** labium **D** dorsal view of aedeagus **E** lateral view of aedeagus **F** ventral view of aedeagus **G** spermatheca. Scale bars: 0.5 mm.

##### Distribution.

China (Yunnan); Laos.

##### Remark.

Following the description of Kimoto and Gressitt (1981, especially figs 21b, 23a), we determined the Chinese specimens as *S.laosensis*. This species was originally recorded in Laos.

#### ﻿ Smaragdina oculata

Taxon classificationAnimaliaColeopteraChrysomelidae

Medvedev, 1988, new country record for China

63E2200A-098D-56BC-BD83-E938174B2DF6

Figures 15 , 16

Smaragdina oculata Medvedev, 1988b: 471 (type locality: Assam, Kaziranga; type deposited: LM); Medvedev 2010: 268.

##### Material examined

**(*n* = 4). China: Yunnan province**: 1 male, 1 female, Mangshi, 15.V.1955, coll. B. Popov (IZ-CAS); 1 male, Mangshi, 17.V.1955, coll. Xingchi Yang (IZ-CAS); 1 male, Mangshi, 17.V.1955, coll. Krejanovsky (IZ-CAS).

**Figure 15.**
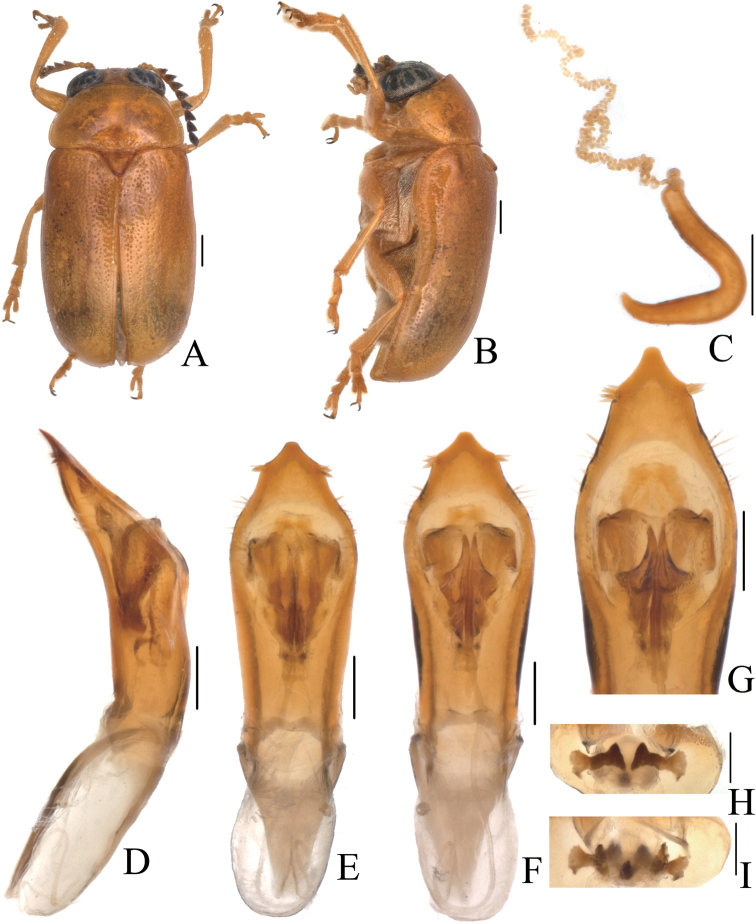
*Smaragdinaoculata* Medvedev, 1988 **A** habitus **B** lateral view of habitus **C** spermatheca **D** lateral view of aedeagus **E** ventral view of aedeagus **F** dorsal view of aedeagus **G** apex of aedeagus **H** ventral rectal sclerites **I** dorsal rectal sclerites. Scale bars: 0.5 mm (**A, B**), 0.2 mm (**C–I**).

##### Measurements

**(*n* = 4).** Body length males: 4.8–5.0 mm, females: 5.5 mm.

**Figure 16.**
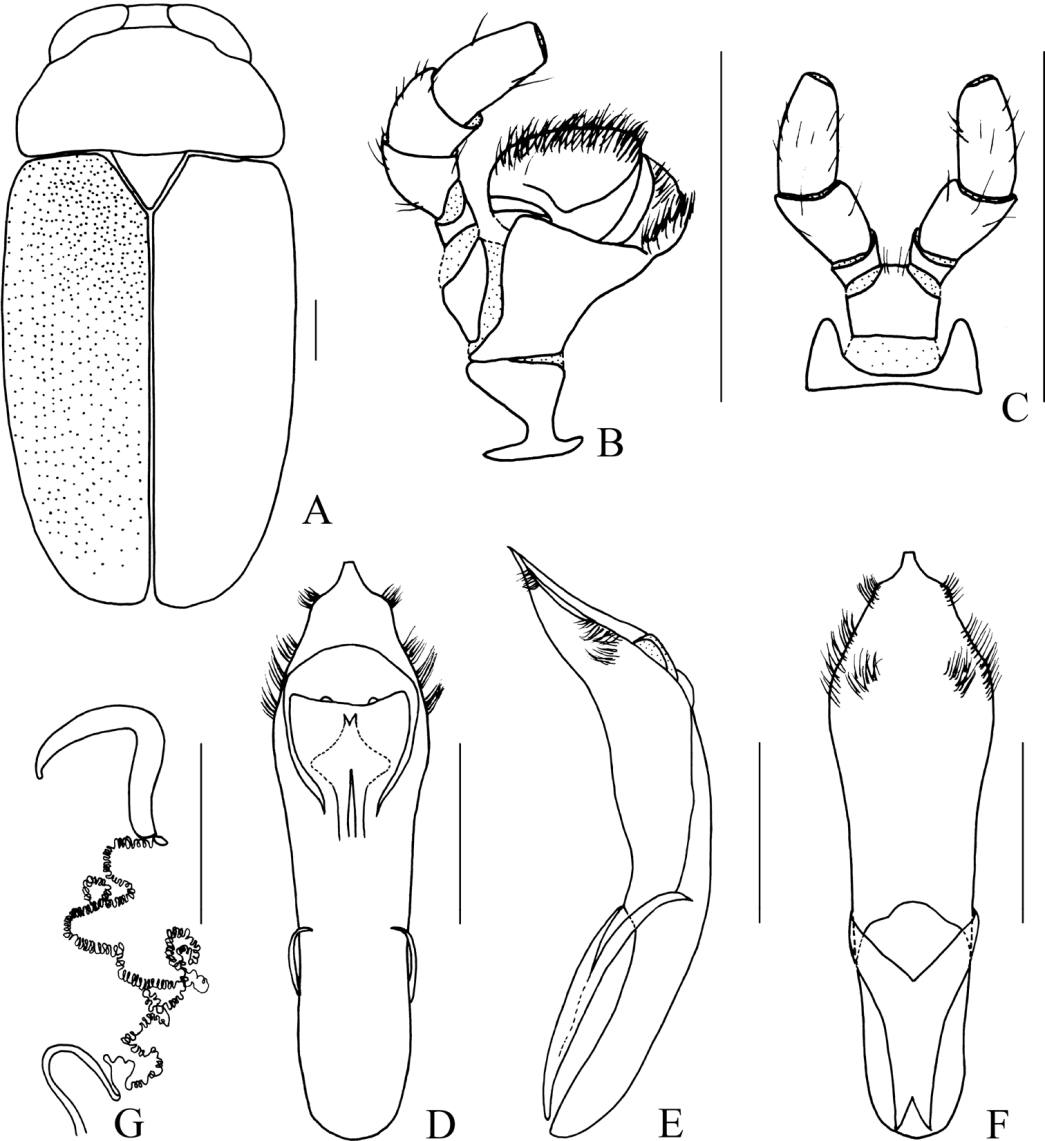
*Smaragdinaoculata* Medvedev, 1988 **A** habitus **B** maxilla **C** labium **D** dorsal view of aedeagus **E** lateral view of aedeagus **F** ventral view of aedeagus **G** spermatheca. Scale bars: 0.5 mm.

##### Distribution.

China (Yunnan); India.

##### Remark.

According to the aedeagus with triangular apex and specific pubescence, the Chinese specimens are certainly *S.oculata* (Medvedev 2010: fig. 82), which has only been recorded from India.

## ﻿Discussion

This study contributes new faunistic discoveries and increases the number of Chinese species of *Smaragdina* from 64 to 72. This is a very important advance in the taxonomy of this large genus in the tribe Clytrini. The Chinese *Smaragdina* have not been studied in last 30 years or longer (Gressitt and Kimoto 1961; Tan et al. 1980; Kimoto and Gressitt 1981; Tan 1988), except for one study originating from our laboratory (Wang and Zhou 2013). This is our second contribution, and it is of considerable value, as it adds to the data published by Medvedev (1988a: a systematic revision of Indo-China *Smaragdina*; 2010: a key to oriental species).

In the early studies on the leaf beetles, including *Smaragdina*, species were reported by older conventions without specimen dissections; thus, few morphological details of aedeagus, spermatheca, rectal sclerites, etc. were provided (Gressitt and Kimoto 1961; Tan et al. 1980; Kimoto and Gressitt 1981). All our papers on leaf beetles, including this and the previous on the Chinese *Smaragdina*, include morphological dissections and provide the detailed color figures of the new and other species (Wang and Zhou 2011, 2012, 2013, 2020; Su and Zhou 2017; Duan and Zhou 2021; Duan et al. 2021a, 2021b). This improvement in methodology dramatically decreases mistakes in species identifications and robustly grounds our discoveries. This, together with our other studies on leaf beetles, are of great significance in filling gaps in the Chinese faunistic composition and promoting the taxonomic progress in the subfamily Cryptocephalinae.

## Supplementary Material

XML Treatment for Smaragdina hejingensis

XML Treatment for Smaragdina magnipunctata

XML Treatment for Smaragdina divisa

XML Treatment for Smaragdina insulana

XML Treatment for Smaragdina kimotoi

XML Treatment for Smaragdina laboissierei

XML Treatment for Smaragdina laosensis

XML Treatment for Smaragdina oculata
